# Bottlenose dolphin (*Tursiops truncatus*) immortalized fibroblasts on novel 3D *in vitro* collagen-free scaffolds

**DOI:** 10.1371/journal.pone.0304992

**Published:** 2024-06-11

**Authors:** Lucrezia Ferretti, Valentina Moccia, Cinzia Centelleghe, Andrea Venerando, Monica Dettin, Elisabetta Sieni, Annj Zamuner, Federico Caicci, Massimo Castagnaro, Valentina Zappulli, Sandro Mazzariol

**Affiliations:** 1 Department of Comparative Biomedicine and Food Science, University of Padua, Legnaro, Italy; 2 Department of Agrifood, Environmental and Animal Sciences, University of Udine, Udine, Italy; 3 Department of Industrial Engineering, University of Padova, Padova, Italy; 4 Department of Theoretical and Applied Sciences, Insubria University, Varese, Italy; 5 Department of Civil, Environmental, and Architectural Engineering, University of Padova, Padova, Italy; 6 Department of Biology, University of Padova, Padova, Italy; University of Naples Federico II: Universita degli Studi di Napoli Federico II, ITALY

## Abstract

Dolphins, as apex predators, can be considered relevant sentinels of the health of marine ecosystems. The creation of 3D cell models to assess *in vitro* cell-to-cell and cell-to-matrix interactions in environmental-mimicking conditions, is of considerable interest. However, to date the establishment of cetacean 3D culture systems has not yet been accomplished. Thus, in this study, different 3D systems of bottlenose dolphin (*Tursiops truncatus*) skin fibroblasts have been analyzed. Particularly, novel scaffolds based on hyaluronic acid and ionic-complementary self-assembling peptides such as RGD-EAbuK and EAbuK-IKVAV have been compared to Matrigel. Histological and fluorescent staining, electron microscopy (TEM) analyses and viability assays have been performed and RT-PCR has been used to detect extracellular matrix (ECM) components produced by cells. Results showed that Matrigel induced cells to form aggregates with lower viability and no ECM production compared to the novel scaffolds. Moreover, scaffolds allowed dispersed cells to produce a collagenous ECM containing collagen1a1, laminin B1 and elastin. The HA-EAbuK-IKVAV scaffold resulted in the most suitable 3D model in terms of cell quantity and viability. The development of this innovative approach is the first step towards the possibility to create 3D *in vitro* models for this protected species.

## 1. Introduction

Nowadays cetaceans’ health is threatened by several natural and anthropic factors and, considering their role in the marine food chain and their longevity, their health might reflect the status of the marine ecosystem [1, 2]. Despite this increasing interest and need of information, several data on their biology and medicine are still missing due to the limitation of studies conducted in their natural environment. Consequently, there is a growing need of developing cetaceans’ study models [3]. Marine mammal cell culturing systems are considered rare multifunctional instruments for acquiring knowledge about the cell physiology and biochemistry of these animals as well as on the damaging effects of anthropogenic and natural toxicants [4, 5]. Several cetacean cell types from multiple tissues have been isolated, including dermal fibroblasts [6–10], bronchial and muscle cells [4], alveolar macrophages [11], primary epithelial cells [12], kidney cells [13, 14], blood cells [15] and cells from mesentery, lung, heart, liver, brain, spleen, thyroid, urinary bladder, periorbital soft tissue, and testes [16]. However, the use of cetacean primary cells for *in vitro* studies have been jeopardized due to their limited doubling capacity and their short life span. Nevertheless, immortalization strategies that have a crucial role in extending cell culture maintenance have been rarely applied to marine mammals’ cells [8, 9]. Among the numerous resident cetacean species in the Mediterranean Sea, the bottlenose dolphin (*Tursiops truncatus*) is one of the most numerous, widespread and charismatic [17]. Furthermore, this species is insert in the Habitats Directive (Council Directive 92/43/EEC), which aims to protect over a thousand species, including mammals, reptiles, amphibians, fish invertebrates, and plants, and more than 200 characteristic habitat types [18]. Bottlenose dolphins inhabit a wide variety of habitats including continental shelf waters, lagoons and enclosed seas, and the waters surrounding islands and archipelagos and strandings are homogeneously distributed along the Italian coastline, giving to the scientific community the opportunity to use these animals as bioindicators of the marine environmental health [19]. Moreover, the use of cell cultures of these species, which *in vivo* experiments are not allowed, facilitates deeper analysis by creating environments that closely mimic the natural tissues where cells’ interactions occur [4]. This could allow to observe how different factors, such as environmental pollutants, influence disease resistance or susceptibility in dolphins, which is critical for managing the health status and conservation of wild populations [4]. For all these reasons, this species is the best candidate to collect tissue samples from freshly dead animals and proceed with cell culture establishment procedures.

With regards to *in vitro* cell models, two-dimensional (2D) cultures, in which cells grow in flat surfaces, are the most common research models [20] both in human and veterinary medicine [21]. Despite their simplicity and low-cost maintenance, 2D cultures suffer disadvantages such as the loss of tissue-specific architecture and the lack of diverse cellular phenotype and proper cell-to-cell and cell-to-matrix interactions [21]. Hence, they are now considered relatively poor models to mimic the natural and complex three-dimensional (3D) structure of tissues [22]. In fact, in the tissues, cells grow within an extracellular matrix (ECM) consisting of a complex architecture of structural, fibrous proteins such as fibronectin, collagen, and laminin embedded in a highly hydrated gel-like material of glycosaminoglycans, proteoglycans, and glycoproteins. This interwoven fiber meshwork provides biochemical and physical signals among cells and compose part of a specific and markedly different 3D microenvironment per each cell type [23]. For these reasons, a variety of 3D culture systems, in which cells grow into 3D aggregates using a scaffold/matrix or in a scaffold-free manner to better resemble original tissues, have been established both in human and veterinary medicine [24, 25]. To date, in human research, 3D culture models such as multicellular spheroids, scaffolds, organoids, organs-on-chips, and 3D bioprinting have been used in toxicology, developmental and stem cell biology, regenerative medicine, drug discovery and in the study of disease mechanisms [26, 27]. Within 3D cell culture systems, scaffolds either of synthetic or biologically-derived materials, have been tested and used as suitable ECM analogues [28, 29]. These substrates are made of materials with different porosity, permeability, surface chemistry, and mechanical characteristics, arranged to provide suitable microenvironments for optimal cell growth and function [21]. For what concerns synthetic scaffolds, Polyethylene glycol (PEG), polyvinyl alcohol (PVA), polylactide-co-glycolide (PLG), and polycaprolactone (PLA) are the most commonly used materials. Instead, those biologically derived include commercially available products such as Matrigel (a reconstituted basement membrane derived from the polymerization of extracts from the Engelbreth-Holm-Swarm mouse sarcoma [30]), proteins, ECM components (collagen, fibrin, hyaluronic acid) and other materials such as chitosan, alginate, or silk fibrils [23, 26, 27]. More recently, complementary ion self-assembling peptides (SAPs) have been discovered as a class of peptides capable of spontaneously forming fibrous structures in the presence of positive monovalent ions [31]. The precursor of this class is the EAK 16-II peptide, which presents the alternation between hydrophobic and hydrophilic amino acids and a charge distribution that alternates between two positively charged side chains and two negatively charged side chains (module II). SAP hydrogels with 99% of water have been proposed as scaffolds for the growth of bone, nervous, cartilaginous, and cardiac tissue [32, 33]. Since only a few attempts have been carried out for the establishment of cetacean 3D culture models [34], the main aim of this study was to develop novel *in vitro* 3D culture systems seeding bottlenose dolphin (*Tursiops truncatus*) immortalized fibroblasts within hyaluronic acid sponge and within hyaluronic acid sponge cross-linked with SAPs conjugated to adhesive sequences of laminin and fibronectin. These scaffolds were compared with the more commonly used Matrigel.

## 2. Materials and methods

### 2.1. Cell line

The cell line used in this study, immortalized fibroblasts, derived from bottlenose dolphin’s dorsal skin samples, collected during post-mortem examination from an adult male stranded in January 2019 along the Veneto coastline, North Adriatic Sea, (Italy). The cited cell line was previously established and patented by the Department of Comparative Biomedicine and Food Science, at the University of Padua (patent n° IT102020000003248-WO2021/16521; https://www.knowledge-share.eu/en/patent/sea-sentinel-system-for-environmental-studies/) [8]. Cells were cultured in Dulbecco’s Modified Eagle Medium/Nutrient Mixture F-12 (DMEM/F-12, Thermo Fisher Scientific, Waltham, MA, USA) supplemented with 20% FBS (Thermo Fisher Scientific, Waltham, MA, USA), 1% penicillin/streptomycin (Corning, New York, NY, USA) and 1% MEM Non-Essential Amino Acids Solution (Thermo Fisher Scientific, Waltham, MA, USA). Cell lines were regularly tested and confirmed to be mycoplasma-free (Mycoalert Mycoplasma Detection Kit, LONZA, Basel, Switzerland).

### 2.2. Materials

Hyaluronic acid (MW = 100–1250 kDa) was purchased from Contipro Biotech S.r.o (Dolni Dobrouc, Czech Republic). Acetonitrile, triethoxysilane (TES), and 1-Ethyl-3-(3-dimethylaminopropyl) carbodiimide (EDC) were from Sigma Aldrich (Steinheim, Germany). Ethanol was obtained from VWR Chemicals Prolab (Fontenay-sous-Bois, France). The Rink Amide MBHA resin, all 9-fluorenylmethoxycarbonyl (Fmoc) protected amino acids and the coupling reagents 2-(1H-benzotriazole-1-yl)-1,1,3,3-tetramethyluronium hexafluorophosphate (HBTU) and Ethyl cyano(hydroxyimino)acetate (Oxima pure) were acquired from Novabiochem (Merck KGaA, Darmstadt, Germany). N,N-dimethylformamide (DMF), trifluoroacetic acid (TFA), N-methyl-2-pyrrolidone (NMP), dichloromethane (DCM), N,N-diisopropylethylamine (DIEA), and piperidine were purchased from Biosolve (Leenderweg, Valkenswaard, The Netherlands).

### 2.3. Self-assembling peptides

#### 2.3.1 Synthesis

The SAPs employed in this study are two analogs of EAbuK 16 module II peptide [35]. EAbuK-16 module II is a 16-mer that alternates pairs of negatively charged (glutamic acid, E) and positively charged (lysine, K) residues. Furthermore, each polar amino acid is separated by a hydrophobic amino acid (Abu α-aminobutyric acid) from the subsequent polar amino acid (EAbuK sequence: H-Abu-Glu-Abu-Glu-Abu-Lys-Abu-Lys-Abu-Glu-Abu-Glu-Abu-Lys-Abu-Lys-NH_2_). The analogs of EAbuK used in this study show the condensation at the C-terminus of EAbuK sequence of (i) the Laminin sequence IKVAV (EAbuK-IKVAV) or (ii) the Fibronectin sequence RGD (RGD-EAbuK). EAbuK-IKVAV was synthesized as reported in [36]. Briefly, Peptide EAbuK-IKVAV (sequence: H-Abu-Glu-Abu-Glu-Abu-Lys-Abu-Lys-Abu-Glu-Abu-Glu-Abu-Lys-Abu-Lys-Ile-Lys-Val-Ala-Val-NH_2_) was synthesized with a Mod. Syro I (MultiSynthec, Witten, Germany) synthesizer using fluorenyl-9-methoxycarbonyl (Fmoc) solid phase chemistry. The synthesis was carried out on a 0.125 mmol of rink amide MBHA resin (0.7 mmol/g) using 5 equivalents of side-chain protected Fmoc amino acids, and 2-(1H-benzotriazol-1-yl)-1,1,3,3-tetramethyluronium hexafluorophosphate (HBTU)/1-hydroxybenzotriazole (HOBt) solution (1:1). The following side chain protections were used: *tert*-butyl ester (OtBu) for Glu; *tert*-butyloxycarbonyl (Boc) for Lys. The first three amino acids and the last sixteen amino acids were introduced through double couplings. After Fmoc deprotection, crude peptide was detached from the resin and protecting groups were released using a 95% trifluoroacetic acid, 2.5% triethylsilane, 2.5% water mixture over 90 min, under magnetic stirring. The resin was filtered off and the solution was concentrated. The crude peptide was precipitated with cold diethyl ether. Purification of the crude product was performed through reverse phase-high performance liquid chromatography (RP-HPLC) using the following conditions: Nova Pak C_18_ semipreparative column (6 μm, 60 Å, 7.8 × 300 mm, Waters, Milford, MA, USA); eluent A: 0.05% trifluoracetic acid (TFA)/water; eluent B, 0.05% TFA/CH_3_CN; gradient, from 5 to 30% of B over 30 min; detector, 214 nm; flow rate, 4 ml/min. RGD-EAbuK (sequence: H-Abu-Glu-Abu-Glu-Abu-Lys-Abu-Lys-Abu-Glu-Abu-Glu-Abu-Lys-Abu-Lys-Arg-Gly-Asp-NH_2_) was synthesized with Fmoc chemistry and a Rink Amide MBHA resin (0.52 mmol/g; scale 0.125 mmol) using a Syro I synthesizer (Multisynthec, Witten, Germany). Five equivalents of side-chain protected Fmoc amino acids, and HBTU/HOBt solution (1:1) were used for each coupling. The side-chain protecting groups were: Boc for Lys, OtBu for Asp and Glu and, 2,2,4,6,7-pentamethyldihydrobenzofuran-5-sulfonyl (Pbf) for Arg. The loading and the couplings from the fifth and the nineteenth step were double. The Fmoc protection of the last attached amino acid was removed, the resin was extensively washed with DCM, and dried for 1 h under vacuum. The peptide was cleaved from the solid support with contemporary side-chain deprotection using the following mixture: 95% TFA, 2.5% TES, and 2.5% MilliQ water (5 mL of total volume) over 90 min, under magnetic stirring. Eventually, the resin was filtered, and the reaction mixture was concentrated. The crude peptide was precipitated with cold diethyl ether. The peptide was used as crude because the mass analysis showed irrelevant side-products.

#### 2.3.2 Mass spectrometry analyses

The identity of the purified EAbuK-IKVAV was confirmed by mass spectrometry (Matrix Assisted Laser Desorption Ionization–Time of Flight AB SCIEX MALDI-TOF 4800 Plus): theoretical mass = 2239.0 Da; experimental mass = 2236.9 Da. MALDI-TOF mass spectrometry confirmed the identity of RGD-EAbuK (theoretical mass = 2054.6 Da; experimental mass = 2055.2 Da).

### 2.4. HA-EAbuK-IKVAV and HA-RGD-EAbuK 3D-scaffolds preparation

EAbuK-IKVAV (7.2 mg) and HA (144 mg), from now on referred to as “SHE”, were dissolved in 12 mL of MilliQ water under magnetic stirring. The solution was divided and weighed into the wells of a 48-well tissue culture plate (320 mg for each sample), frozen in liquid nitrogen, and lyophilized. The scaffolds were cross-linked with 60 mM EDC in 95% ethanol solution for 24h. The scaffolds were washed both with Ethanol (6 times each) and MilliQ (6 times each) in an ultrasound bath for 1 min, and another 2 min not sonicating. Eventually, the scaffolds were frozen at −20 °C before final lyophilization. The same protocol (7.2 mg RGD-EAbuK and 144 mg HA in 12 mL MilliQ water; lyophilization; cross-linking with 60 mM EDC in 95% ethanol for 24 h; washings) was carried out for the preparation of HA-RGD-EAbuK matrices (5% w/w RGD-EAbuK/HA), from now on referred to as “SHR2”. On the other hand, the quantity of RGD-EAbuK was lower for the matrices at 2.5% w/w RGD-EAbuK/HA (3.6 mg), from now on referred to as “SHR1”, and higher for the matrices at 10% w/w RGD-EAbuK/HA (14.4 mg), from now on referred to as “SHR3”. The scaffold made only of HA was named “SH”.

### 2.5. Cell viability assay

17.000 cells per well were seeded inside the 3D scaffolds, previously hydrated with 100 μL of Dulbecco’s Modified Eagle Medium/Nutrient Mixture F-12 (DMEM/F-12, Thermo Fisher Scientific, Waltham, MA, USA) supplemented with 20% FBS (Thermo Fisher Scientific, Waltham, MA, USA), 1% penicillin/streptomycin (Corning, New York, NY, USA) and 1% MEM Non-Essential Amino Acids Solution (Thermo Fisher Scientific, Waltham, MA, USA) for 30 minutes before the seeding in a 96 well plate. This medium was then used in all further experiments. For experiments performed on Matrigel, the same quantity of cells within 30 μL of medium was seeded on 60 μL of Matrigel (Corning, New York, NY, USA), previously left to solidify for 30 minutes at 37° in a 96 well plate. A technical triplicate of each type of seeded matrix was performed. After 24h and 72h, a volume of CellTiter-Glo^®^ 3D Cell Viability Assay (Promega, Madison, WI, USA) equal to the volume of cell culture medium was added to each well. After vigorous mixing for 5 minutes, the plates were incubated at room temperature for 25 minutes. Two different collecting conditions were tested for cell viability assay. In the first one, both cells in-suspension within the matrix and cells adherent to the bottom of the well, carefully mechanically scraped, (from now on referred to as “bottom condition”) were collected and transferred along with the cell viability reagent into opaque-walled multiwell plates. In the second one, only cells in-suspension within the matrix without collecting the cells adherent to the bottom (from now on referred to as “no bottom condition”) were measured. Luminescence was measured with the multilabel plate reader VICTOR^™^ X4 (PerkinElmer^®^). The mean luminescence intensity detected is proportional to the ATP quantity present in the sample, which is the marker for the presence of metabolically active cells. Raw data and significant p-values of the CellTiter-Glo^®^ 3D Cell Viability Assay are reported in S1 and S2 Tables.

### 2.6. Fluorescent staining

For fluorescence imaging, 400.000 cells per well were seeded in 6-wells plate and cultured for 4 days. Then, the medium was removed and cells were gently washed with PBS before being incubated for 5 minutes with Hoechst 33342 (1:2000) (Thermo Fisher Scientific, Waltham, MA, USA) and 20 minutes with CellBrite^®^ Cytoplasmic Membrane (1:5000) (Biotium, Fremont, CA, USA) to stain nuclei and plasma membrane respectively. After staining, cells were washed with PBS, harvested by trypsinization and were pelleted by centrifugation at 250 g for 5 minutes (centrifuge REMI R-10M). Then, scaffolds previously hydrated with 150 μL of medium for 30 minutes in a μ-Slide 8 Well high ibiTreat (Ibidi, GmBH, Germany) were seeded with 51.000 stained cells in 50 μL of growth medium. The 3D scaffolds were gently pierced with the tip containing the cell suspension to allow cell penetration. After 1 h incubation, 100 μL of culture medium was added to all the seeded scaffolds. In the same μ-Slide system, the same quantity of stained cells was seeded within 100 μL of Matrigel, previously left to solidify for 30 minutes at 37°, and within 300 μL of medium in the case of the 2D samples. At 24h and 72h from seeding, the stained cultures were observed and images were acquired with both inverted epifluorescence microscope (Olympus IX51) and Leica TCS SP5 confocal microscope equipped with Leica HC PL FLUOTAR 20x/0,50 objective. All the images were analyzed using ImageJ software.

### 2.7. Histological staining

#### 2.7.1. Scaffolds seeding

Round coverslips (Thermo Fisher Scientific, Waltham, MA, USA) were positioned on the wells of a 24 well plate and were coated with 2% gelatine from bovine skin (Sigma-Aldrich, Steinheim, Germany). The scaffolds were hydrated onto the coverslips with 200 μL of culture medium per well for 30 minutes before being seeded with 100.000 cells per well. At intervals of 1h, 200 μL and subsequently 300 μL of culture medium per well were added, up to a total volume of 1 mL per well. A technical triplicate of each type of seeded, unconditioned (without cells) matrix and 2D samples was performed.

#### 2.7.2. Scaffolds fixation and staining

After 24h, 72h, and 7 days from the seeding, the culture medium, including the scaffold, was removed by gentle aspiration from the top of each coverslip. The coverslips were then fixed with 4% formaldehyde for 70 minutes, and then mounted on microscope slides by Eukitt mounting medium. The same procedure was performed on unconditioned scaffolds used as negative control and on 2D cell culture. Finally, the slides were stained with Hematoxylin and Eosin (HE, Diapath, Martinengo, BG, Italy) and Masson trichrome (MT, Bio-Optica, Milano, MI, Italy) techniques. Cells were photographed using an inverted microscope (Olympus IX50). Due to its chemical-physical characteristics, it was not possible to use the same histological technique with Matrigel without damaging the adherent cells.

### 2.8. Transmission electron microscopy (TEM)

Scaffolds were prepared and seeded, as described above for the histological staining, in a 24 well suspension plate, but no coverslips were used. Similarly, 200 μL of Matrigel, previously left to solidify for 30 minutes in the incubator, were seeded with 100.000 cells per well. A technical triplicate of each type of seeded, unconditioned matrix and 2D samples was performed. Differently from histology, in this case, it was possible to fix the adherent cells of both scaffolds and Matrigel systems, due to the different experimental protocol. After 24h, 72h and 7 days, the culture medium was removed, and cells were fixed with 2.5% glutaraldehyde in 0.1 M sodium cacodylate buffer (pH 7.4) at 4 °C. Samples were post-fixed with a mixture containing 1% osmium tetroxide and 1% potassium ferrocyanide in 0.1 M sodium cacodylate buffer for 1h at 4 °C. After 3 washes with water, samples were dehydrated by immersion in increasing concentrations of ethanol and embedded in epoxy resin (Sigma-Aldrich, Steinheim, Germany). Ultrathin sections (60–70 nm) of the well were obtained with an Ultrotome V (LKB) ultramicrotome, counterstained with uranyl acetate and lead citrate. Samples were observed with a Tecnai G 2 (FEI) TEM operating at 100 kV and images were acquired with a Veleta digital camera (Olympus Soft Imaging System).

### 2.9 Reverse transcription polymerase chain reaction (RT-PCR)

Scaffolds were seeded as described above in a 24 well plate. A technical triplicate of each type of seeded, unconditioned matrix, and 2D samples was performed. At 72h and 7 days from seeding, cells were harvested by trypsinization and pelleted by centrifugation at 250 g for 5 minutes. Then, total RNA was extracted using RNeasy Plus Mini Kit (Qiagen, Hilden, Germany) according to the manufacturer’s instructions and quantified using both NANODROP 2000 (Thermo Fisher Scientific, Waltham, MA, USA) and QUBIT RNA BR Assay Kit (Thermo Fisher Scientific, Waltham, MA, USA). RNA of bottlenose dolphin skin, extracted using the same Kit, was used as tissue positive control, while RNA from unconditioned scaffolds as blank. Primers for collagen, laminin, and elastin detection were designed using Primer3web (https://primer3.ut.ee/) and purchased from Invitrogen, Waltham, MA, USA. Their sequences are reported in Table 1.

**Table 1:** Primers for RT-PCR amplification used in this study.

Gene	Primer sequence	Fragment length, bp
GAPDH	F-CAAGGCTGTGGGCAAGGTCATC R-TTCTCCAGGCGGCAGGTCAG	22
Collagen type I, alpha-1 (COL1A1)	F-CCAGCCACCTCAAGAGAAGG R-ACATCTTGAGGTCACGGCAG	188
Collagen type I, alpha-1 (COL1A1)	F-GAGAGAGGTGAACAAGGCCC R-AAACCTCTCTCGCCTCTTGC	155
Laminin subunit beta 1 (LAMB1)	F-GGAGGGGTGTGTGATGAGTG R-TTACACCGACACTGACCAGC	212
Laminin subunit beta 1 (LAMB1)	F-ATGGTTCACGGACACTGCAT R-CACTCATCACACACCCCTCC	218
Elastin (ELN40)	F-TTGGTGAGTTGCTCCCGATG R-CAGATGTGGGTGAGGACGAG	203

GAPDH was chosen as the housekeeping gene (Invitrogen, Waltham, MA, USA). The RevertAid First Strand cDNA Synthesis Kit (Thermo Fisher Scientific, Waltham, MA, USA) was used to synthesize cDNA. Primers and RNA of scaffolds and 2D samples were incubated at 42°C for 60 min followed by 70°C for 5 min. Amplification was performed using GoTaq(R) G2 DNA Polymerase (Promega, Madison, WI, USA) with the following PCR conditions: an initial denaturation step at 95°C for 2 min; 30 cycles of 30 s at 95°C, 30 s at 60°C, 1 min at 73°C; and an extension step at 73°C for 5 min. The procedure was carried out using SimpliAmp Thermal Cycler (Thermo Fisher Scientific, Waltham, MA, USA). Products of the PCR reaction were screened on a 2% agarose–Tris-acetate-EDTA (TAE) gel using Sybr safe DNA gel stain (Invitrogen, Waltham, MA, USA). The images of the gel were captured with iBright instrument (Thermo Fisher Scientific, Waltham, MA, USA).

### 2.10. Statistical analysis

Statistical analyses were performed with GraphPad Prism 8 software. Differences between more than two groups were tested with multiple comparison with one way ANOVA followed by Kruskal-Wallis. Level of significance was set at p <0 .05. Data were expressed as mean ± SD.

## 3. Results

### 3.1. Cell viability

**Fig 1:**
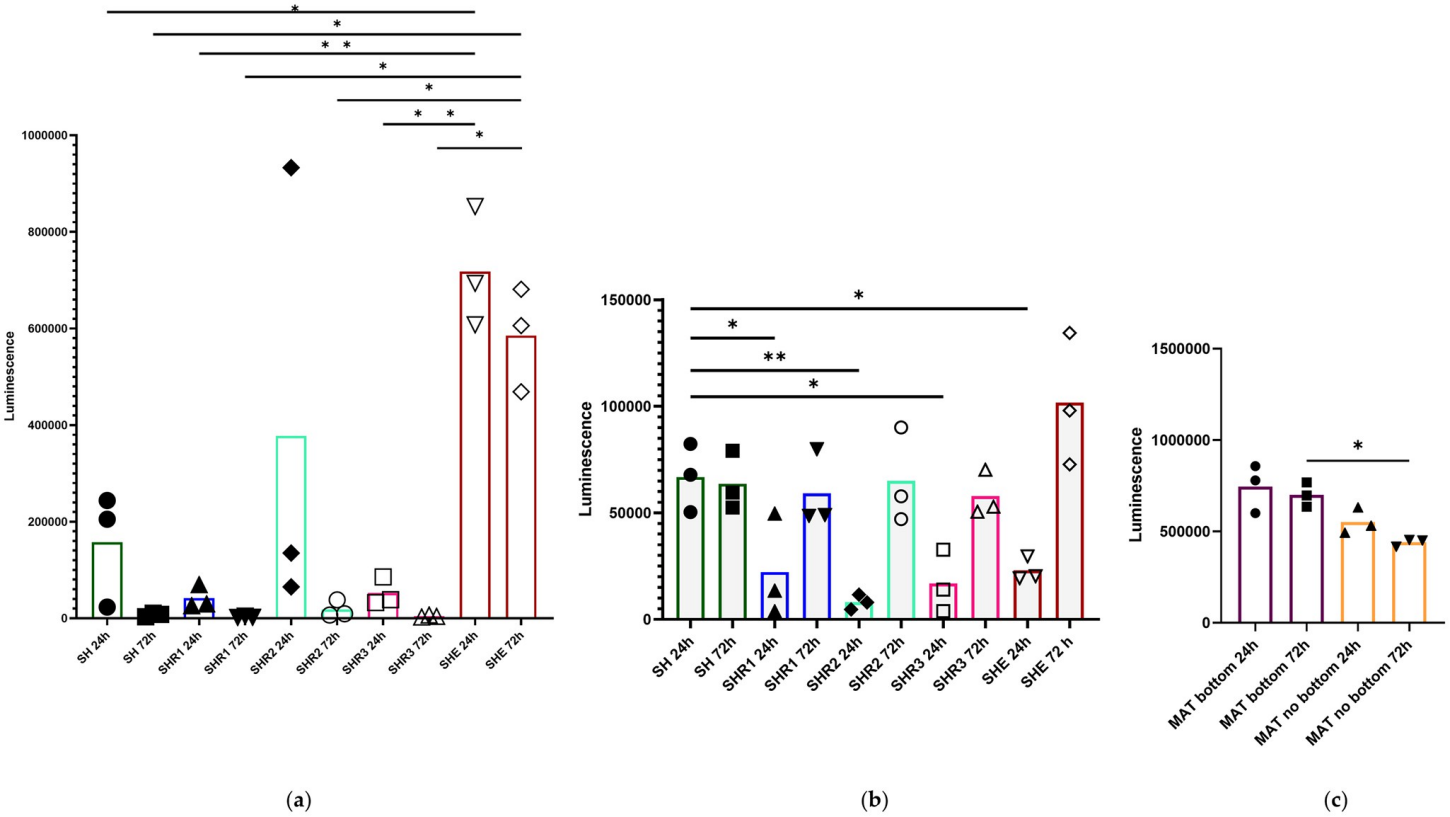
CellTiter-Glo^®^ 3D Cell Viability Assay on tested scaffolds and Matrigel. CellTiter-Glo^®^ 3D Cell Viability Assay at 24h and 72h from cell seeding on tested hyaluronic acid sponge cross-linked with SAPs conjugated to adhesive sequences of laminin and fibronectin scaffolds in bottom condition (a) and in no bottom condition (b) as well as on Matrigel in both conditions (c). HA shown as SH; 2.5% RGD-EAbuK/HA as SHR1; 5% RGD-EAbuK/HA as SHR2; 10% RGD-EAbuK/HA as SHR3; EAbuK-IKVAV/HA as SHE. Differences between more than two groups were tested with multiple comparison with one way ANOVA test followed by Kruskal-Wallis. Level of significance was set at p <0 .05; *p < 0.05; **p < 0.01.

Cell viability assay showed that in the bottom condition, therefore of both the in-suspension and adherent cells seeded within all scaffolds, the viability was higher than that measured in the no bottom condition, thus of the in-suspension cells only (compare Fig 1a and 1b), but generally it drastically decreased after 24h with the exception of SHE scaffold, in which the viability dropped but much less markedly (Fig 1a). Conversely, in the no bottom condition, the viability of the in-suspension cells increased from 24h to 72h in SHE, SHR1, SHR2, and SHR3 but not in SH scaffold (Fig 1b). The SHE scaffold showed the greatest viability in both conditions. Particularly, in the bottom condition the SHE viability was noticeably higher than that measured in the other scaffolds and its values were comparable to those obtained in Matrigel. Similarly, also in Matrigel the viability of the cells in the bottom condition was higher than that found in the no bottom condition, however, in both cases it decreased after 24h (Fig 1c).

### 3.2. Cell morphology

**Fig 2:**
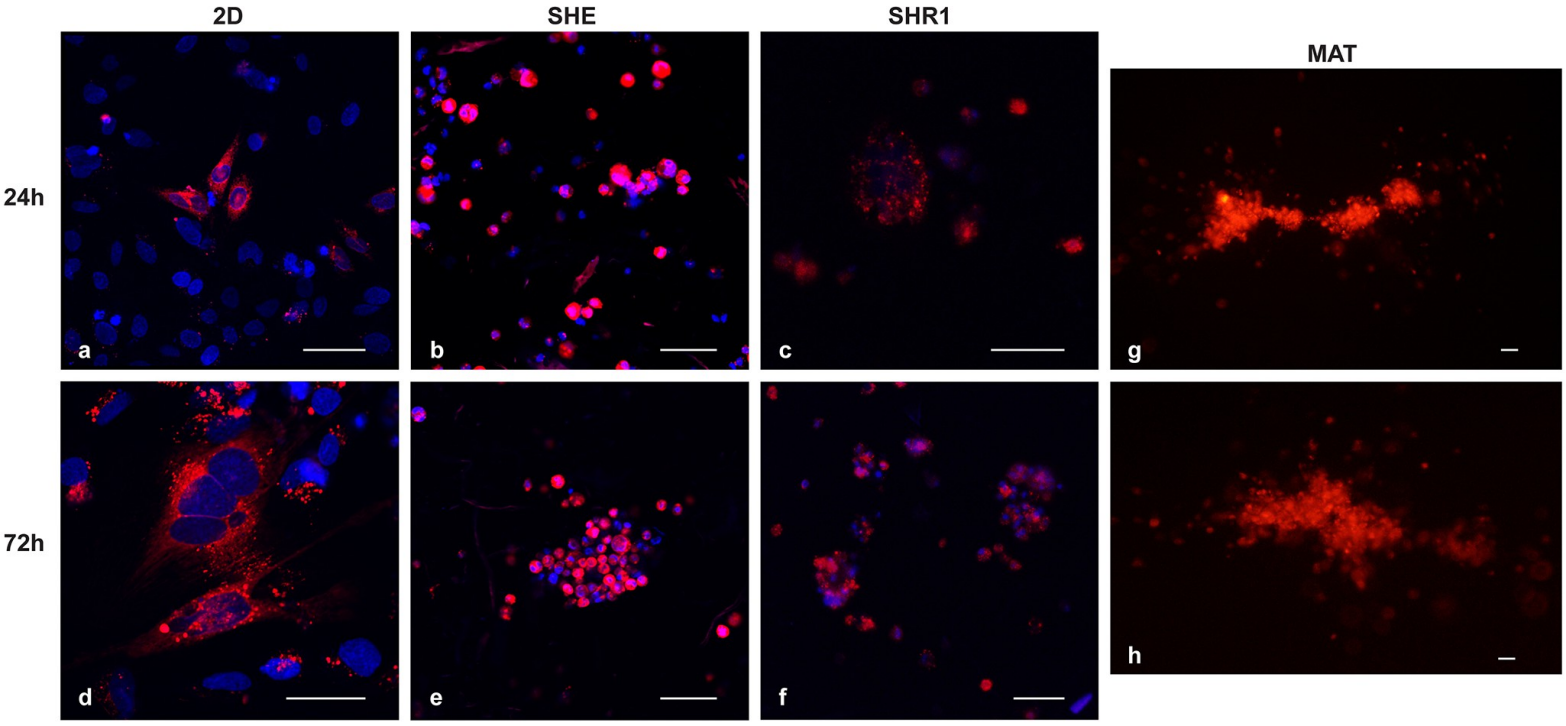
Confocal microscopy of culture grown on 2D system, SHE and SHR1 scaffolds and on Matrigel. Representative confocal microscopy of culture grown on 2D system (a, d), SHE scaffold (b, e), SHR1 scaffold (c, f) and on Matrigel (g, h) at 24h and 72h after seeding. All the figures show a merge of cellBrite and Hoechst staining, 20xair magnification. Bar represents 50 μm.

The confocal microscopy live imaging of the in-suspension cells stained with fluorescent dyes, showed that 3D cultures within scaffolds and Matrigel, compared with a classical 2D culture, had some peculiarities both in cell shape and growth trend. The cells in-suspension within the different 3D matrices had a round or polygonal shape (Fig 2b, 2c, 2e and 2f–2h) (S1 Fig). whereas in the 2D culture they appeared more elongated (Fig 2a and 2d). Moreover, in the scaffold systems at 24h they tended to arrange themselves in single cells, while at 72h they organized into small groups. In Matrigel, instead, cells organized themselves into bigger groups already at 24h, possibly indicating rapid cell growth. In all the samples, the membranes, stained in red in Fig 2, were not sharply demarcated due to the internalization of the dye. Interestingly, in the Matrigel system at 24h post-seeding it was not possible to visualize nuclei since Hoechst dye was metabolized by the cells. On the contrary, it was still visible in the scaffold systems even after 72h, again suggesting that cells proliferated faster in Matrigel.

### 3.3. Cell growth

**Fig 3:**
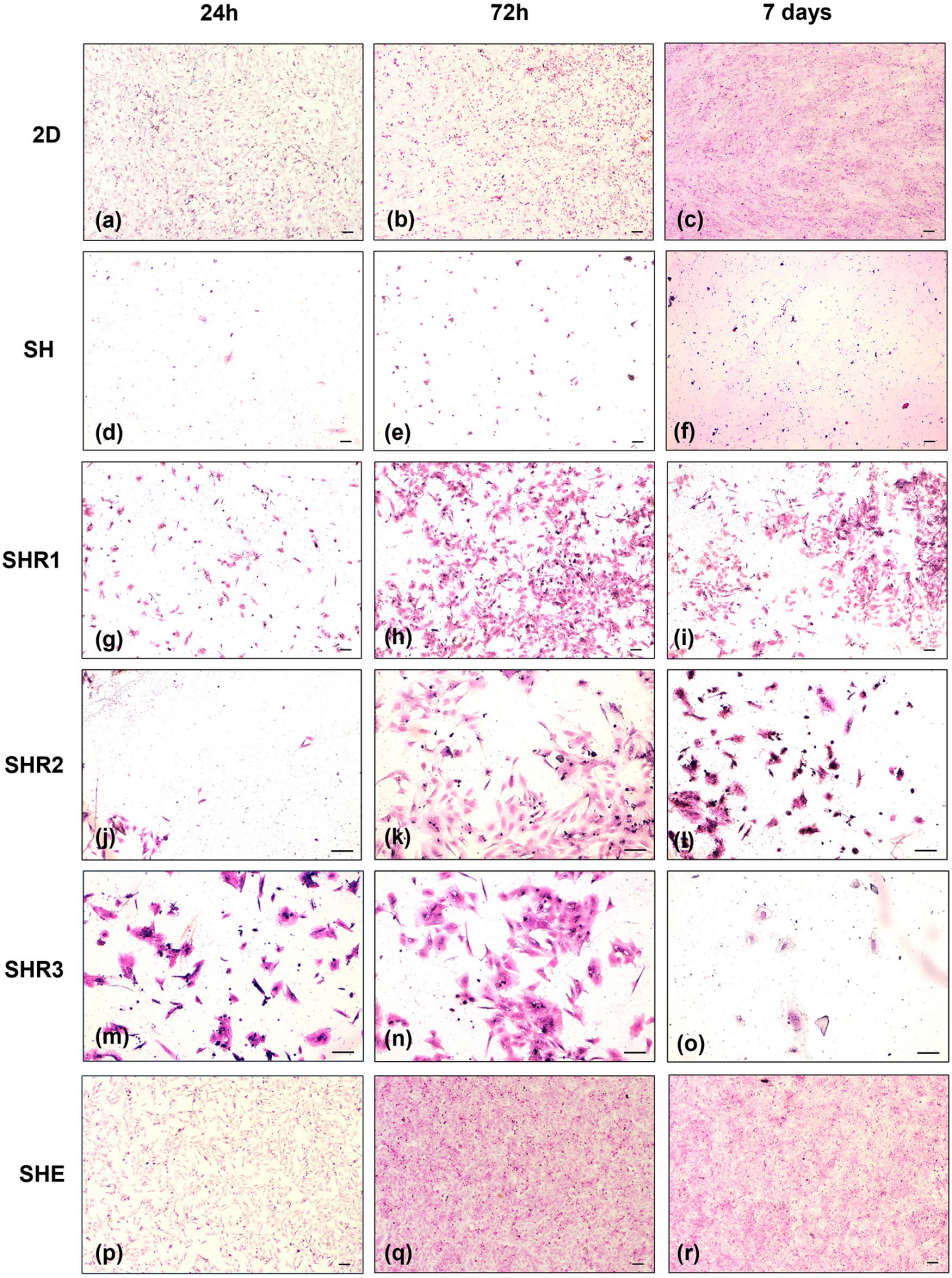
HE images of the adherent cells in the 2D and 3D scaffold systems. HE images of the adherent cells in the 2D (a-c) and 3D scaffold systems (d-r) at different post-seeding times (24h, 72h and 7 days). Cells within SHE (5x magnification) reach 60% confluence at 24h (p) and a 100% confluence at 72h (q). Instead, in SHR1 (5x magnification), SHR2 and SHR3 (10x magnification) the cells barely reached 50% confluence at 72h, then the cellularity decreased between 72h and 7 days (j-o). 2D culture (5x magnification) show a higher cellularity in each time point compared to scaffolds (a-c). SH (5x magnification) was not suitable for cell growth (Fig 3d–3f). Bar represents 100 μm.

Only adherent cells could be evaluated with histology. The HE stain of the scaffold’s fixed adherent cells showed that among scaffolds, SHE was the matrix associated with the fastest cell growth. Indeed, at 24h from seeding, adherent cells within the latter matrix reached a confluence of 60% and at 72h they created a confluent monolayer (Fig 3p–3r). Instead, in SHR1, SHR2 and SHR3 the cells barely reached 50% confluence at 72h and the cellularity decreased between 72h and 7 days (Fig 3g–3o). Furthermore, comparing the HA-RGD-EAbuK scaffolds stained with HE and Masson trichrome staining, SH was not suitable for cell growth (Fig 3d–3f), while SHR1 resulted in the best cell growth (Fig 3g, 3h and 3j). Compared to all the 3D scaffolds, 2D culture showed a higher cellularity in all the time points, with cells forming a confluent monolayer after 72h (Fig 3a–3c).

### 3.4. De novo synthesis of extracellular matrix

**Fig 4:**
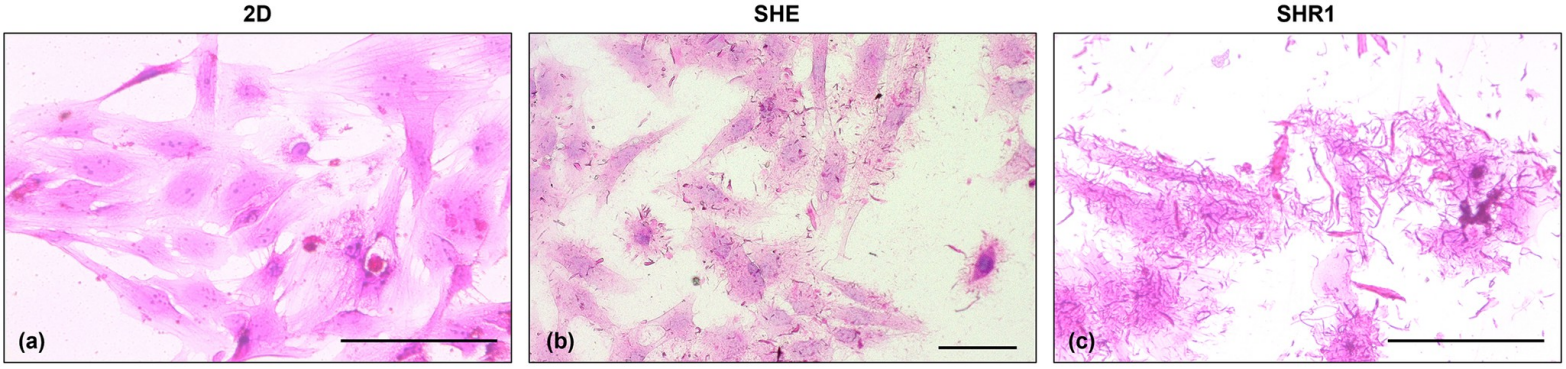
Extracellular filaments of the adherent cells in the 3D scaffold systems. HE images of the adherent cells in the 2D (a) and 3D scaffold systems (b, c) at 72h, 40x magnification (a, c), 20x magnification (b). In both HA-RGD-EAbuK and HA-EAbuK-IKVAV scaffolds, extracellular filaments are visible mostly around cells (black arrow). No filaments were found in the 2D cultures (a). Bar represents 100 μm.

The staining of the scaffold’s adherent cells also allowed us to observe the appearance at 24h of extracellular filaments around the cells that were not detected in the unconditioned scaffolds (S2 Fig) or in the 2D culture (Fig 4a). The extracellular filaments appeared composed of thin filaments that were mainly located near the cells but also in the surrounding area (Fig 4b and 4c). Such structures were found in SHE, SHR1, SHR2 and SHR3 scaffolds, and were not visible in SH scaffolds and in 2D cultures. Masson trichrome staining didn’t specifically stain the extracellular filaments.

### 3.5. Cell ultrastructure analysis by TEM

**Fig 5:**
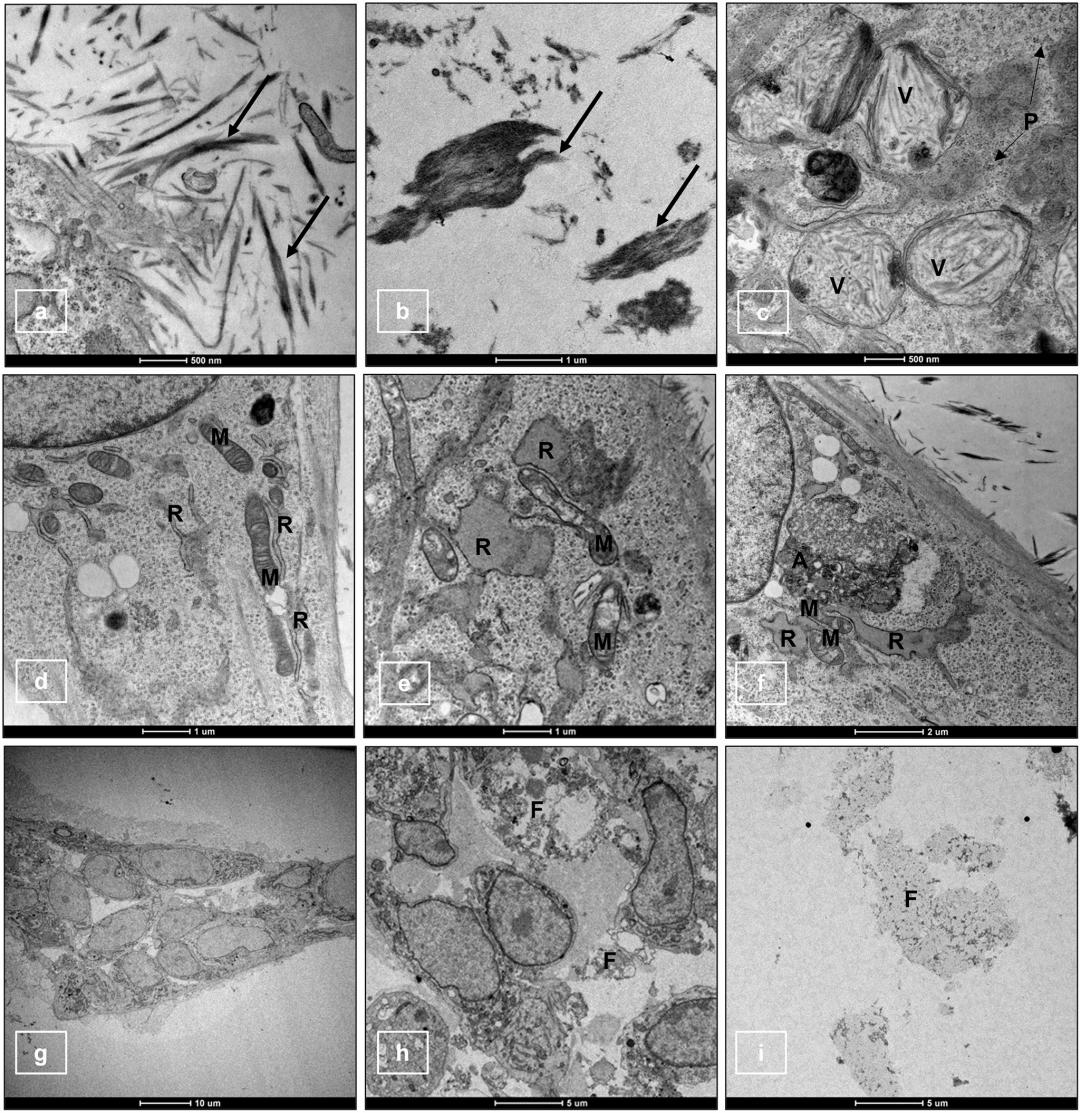
TEM images of adherent cells seeded within scaffolds and Matrigel. Details of TEM images of adherent cells seeded within SHR1 (a, b), SHE (c, d) at 72h from the seeding, adherent cells within SHR2 (e) at 24h and within SHR3 (f) at 7 days. In g, h and i images, details of adherent cells within Matrigel at 24h, 72h and 7 days. In a, b and c images, presence of collagen microfibrils around the cells (black arrows), within intracytoplasmic vesicles (V) and in the surrounding area (black arrow) is visible. In the c image it is also possible to observe polyribosomes (P). Well-developed cisternae of RER (R) and intact mitochondria (M) are shown in image d. Dilated cisternae of RER (R), damaged mitochondria with broken or disrupted cristae (M), and autophagic bodies (A) are visible in e and f images. In the g image, Matrigel cellular aggregate of morphologically intact cells at 24h is shown, while in the h image presence of cellular fragments (F) within the aggregates at 72h can be observed. In the i image only cellular fragments are visible at 7 days.

TEM was used to reveal ultrastructural details of the adherent cells embedded within the different HA/peptide-based scaffolds in comparison with Matrigel. Particularly, we were interested in obtaining a detailed image of the extracellular filaments highlighted by the histochemical analysis. Apart from SH scaffold in which ECM filaments were not evidenced, TEM analysis of the other scaffolds showed the presence of collagen microfibrils with different length and shape in all the other HA/peptide-based scaffolds (S3 Fig). Such ECM filaments were visible at 72h from the seeding around the cells and in less quantity in the surrounding area (Fig 5a and 5b). Interestingly, in SHE and SHR1 it was also possible to evidence the presence of several intracytoplasmic vesicles carrying collagen microfibrils (Fig 5c). The quantity and the shape of the filaments appeared stable from 72h up to 7 days from the seeding. Furthermore, it was possible to observe in all conditions some additional features of the cells. In particular, numerous mitochondria and polyribosomes (Fig 5c), indicative of cells with intense protein synthesis, were present. Noteworthy, after 72h from the seeding in all scaffolds, cells exhibited dilated cisternae of rough endoplasmic reticulum (RER), damaged mitochondria with broken or disrupted cristae (Fig 5d) and a conspicuous number of autophagic bodies indicating cellular suffering. These findings increased at 7 days from the seeding (Fig 5f). On the other hand, TEM analysis of cells within Matrigel at 24h showed intact cells (Fig 5g). However, at this time point, the same signs of cellular suffering observed in the scaffolds at 72h, were present in the Matrigel system. At 72h cells within Matrigel contained cytoplasmic organelles debris (Fig 5h), and at 7 days only cellular fragments were visible in the matrix (Fig 5i). At all the time points, no ECM filaments were found within Matrigel.

### 3.6. Scaffolds ECM biomolecular analysis

To investigate the composition of the filaments observed within scaffolds RT-PCR was carried out. The results showed that both collagen and laminin transcripts were present in all the seeded scaffolds and in the 2D samples at 72h and 7 days from the seeding. Conversely, they were not found in the unconditioned scaffolds. On the other hand, elastin transcripts were found in the 2D samples and in SHR3 at 72h after the seeding, and in SH and SHR1 at 7 days (S4 Fig). No amplification was performed from cells seeded on Matrigel. The original and uncropped gel results are reported in the supportive information (S1 Raw images).

## 4. Discussion

In this study we applied novel 3D *in vitro* systems based on self-assembling peptides (SAPs) scaffolds to grow bottlenose dolphin immortalized fibroblasts and compared them with the well-known Matrigel system and the more commonly used 2D systems [37]. Scaffolds based on both collagen and hyaluronic acid have been already used as 3D ECM models for different aims in human research in order to better mimic the *in vivo* microenvironment with cell-to-cell and cell-to-ECM contacts [27, 38, 39]. Particularly, SAPs scaffolds have been shown to support the cell-to-environment exchange of oxygen, nutrients, bioactive factors, and waste products both in human and veterinary tissue engineering and in human cancer research [34, 40–42]. Although these scaffolds are able to mimic the *in vivo* environment, poor cell adhesion, due to the hydrophilicity of hydrogels, and the lack of cell binding motifs are well-known limitations [23]. These limitations can be overcome by conjugating cell-binding motifs within the scaffolds [43]. In our study we used collagen-free 3D SAPs scaffolds based on crosslinked and lyophilized matrix components, including HA and EAbuK SAPs carrying the Arg-Gly-Asp (RGD) and laminin sequence (IKVAV) adhesion cell-binding motifs [40]. RGD sequence is the known binding domain of fibronectin and was previously reported to support viability and adhesion in human osteoblasts, cardiomyocytes and endothelial cells [44–46]. IKVAV, a small peptide derived from laminin-111, promotes cell adhesion, induce tumor growth, metastasis, activation/secretion of proteases and angiogenesis in humans [40, 47]. In HA sponge with cross-linked complementary ionic self-aggregating peptides, it was observed that tumor cells grew and personalized the matrix with extensive collagen production [38].

To the best of our knowledge, HA sponge cross-linked with SAPs conjugated to adhesive sequences of laminin and fibronectin scaffolds have never been used in veterinary medicine, and it was interesting to test if dolphin fibroblasts could grow similarly to tumor cells, personalizing the matrix with collagen production. To validate these scaffolds as potential models for pathobiology and ecotoxicology studies of marine mammals, we chose to analyze cell viability, morphology, growth and ECM production of bottlenose dolphin (*Tursiops truncatus*) immortalized fibroblast cells within different 3D scaffolds differently composed of HA-RGD-EAbuK, HA-EAbuK-IKVAV, and HA in comparison to the well-established Matrigel.

When assessing the cell viability, the inclusion of also the cells adherent to the bottom of the plate together with the cells in-suspension within the scaffold (bottom condition) obviously showed better results than when assessing only the in-suspension cells. Moreover, among our scaffolds, SHE (HA-EAbuK-IKVAV) was the one with the highest viability, suggesting that the specific combination of SAPs and adhesion motifs in this scaffold might allow a better fibroblasts proliferation. However, the viability of the adherent cells decreased after 24h, while the viability of the cells in-suspension within the scaffolds increased after 24h. Similar results were already obtained by Sieni and colleagues seeding different cell lines within similar scaffolds [39]. This may be explained by the fact that adherent cells, even if in contact with the 3D matrix, might not receive nutrients and oxygen at the same extent as the cells in-suspension, resulting in reduced ATP metabolism. Indeed, it has been demonstrated in human scaffold systems that insufficient nutrient delivery may happen as a result of arrangement of cells in the various planes of the 3D structure [39]. Curiously, with regards to Matrigel, the viability decreased after 24h both for adherent and in-suspension cells. This could be due to the change in metabolism of the cells related to this 3D matrix. In particular, Matrigel appears as poorly suitable to grow fibroblasts [48]. Indeed, Matrigel is a reconstituted basement membrane gel, and this is not the natural extracellular environment of fibroblasts [30, 48]. Hence SAPs scaffolds, and particularly SHE, appeared as a better 3D model for fibroblasts viability when compared to Matrigel.

When it comes to cell morphology, our results showed that the cells in-suspension within scaffolds had oval to round shape and they appeared as single cells at 24h, while they were organized in small groups by 72h. It is well known that *in vitro* fibroblasts shape is influenced by matrix stiffness and cell-matrix adhesions [49]. Cell-matrix adhesions depend on the cell surface structures that mediate cell interactions with ECM and include both focal and fibrillar adhesions. The former are integrin-based structures that mediate strong cell-substrate adhesion, while the latter generate extracellular fibrils of fibronectin [50]. It was found that in low tension states, as for example in a floating 3D matrix, human fibroblasts didn’t show actin stress fibers and matrix biosynthesis and only few focal adhesion phenotypes were observed [51]. Whereas, in stiff matrices, which provide a high-tension state, the fibroblasts started cytoskeletal reorganization to induce the formation of stress fibers and focal adhesions [52]. Fibroblasts differentiation is therefore more variable within a 3D matrix than in 2D hard plastic cell culture plates [52–54]. Indeed, in our 3D models, the cells adherent to the bottom had a spindle-shaped morphology which was more comparable to the morphology of cells in the 2D systems. The presence of the cells adherent to the bottom can be explained by the geometry and the pore size distribution of the tested scaffolds. The stiffness of the matrices was evaluated and the scaffold with EAbuK-IKVAV at 5% appears to have a Young’s modulus of around 350 Pa: it is true that the stiffness differs from that of the soft tissues where the native fibroblasts usually reside (between 800 and 4000 Pa) [55], but it is also true that stiffness favors the increase in focal contacts, increase in cell area and spreading while softer gels favor migration and therefore colonization of the gel in 3D. 3D matrices with similar porosity but considerably different pore geometry (i.e., fibrous versus spherical pores) and size can lead to different mass transport profiles, cell seeding and migration efficiency, and these can influence the ability of cells to pierce the scaffold reaching the bottom [23]. Hence, fibroblasts can spread to the bottom, adopting a similar shape as is observed in 2D culture, but they will also interact with the above 3D matrix from multiple sides. In Smithmyer and colleagues’ study, authors created a system in which human pulmonary fibroblasts were in contact with both the bottom well and a 3D matrix system consisting of hydrogels formed from an 8-arm PEG-norbornene, a di-cysteine cell degradable peptide [49]. In accordance with our results, they demonstrated that these fibroblasts adopted a spread and slightly clustered morphology, different from the rounded morphology of the same cells in-suspension within the 3D matrix [49]. When comparing scaffolds to Matrigel, in Matrigel the cells in-suspension had oval to round shape forming within 24h grape-like spheroids. It has been well demonstrated that several types of animal and human cells tend to create irregularly round aggregates within Matrigel [37, 56–58].

Concerning cellular growth assessed by histology on adherent cells, it was possible to observe that cells proliferated faster in 2D than in the 3D systems. A previous study similarly reported that a multiplicity of cell lines showed reduced proliferation rate in 3D cultures compared to those cultured in 2D [26]. Indeed, cell proliferation is regulated by various pathways that may be differentially regulated depending on the microenvironments. This can be controlled by the molecular signaling derived from the ECM as well as from the adjacent cells [59, 60]. Moreover, in the 2D *in vitro* systems there is often an upregulation of many genes that promote rapid growth, proliferation, as well as those that allow the cells to respond to growth factors in the culture medium [59]. In addition, the adherent cells had a different growth rate among the diverse scaffolds, being SHE the best scaffold for cell growth and SH the scaffold with the lowest adherent cellular growth. This histological evidence is slightly different from the viability assay (see above) by which, when both adherent and in-suspension cells were included (bottom condition), the worst scaffolds were SHR3 and SHR1. Noteworthy, even if the viability of the in-suspension and adherent cells decreased after 24h, histology showed that the adherent cells reached a 100% confluence at 72h. This is related to the fact that, unlike histology, the viability assay is an indirect method to count cells, based on the measurement of cell metabolism changes [61]. Histology also showed in the scaffold’s adherent cells the presence of extracellular filaments around the cells in the majority of scaffolds. They appeared at 24h and increased in quantity over time. These findings were not visible in SH, in the unconditioned scaffolds or in the 2D culture. The possible explanation of these structures is that even just the contact with the scaffolds induced the production of extracellular filaments. These filaments were not assessable in Matrigel, nor in cells in-suspension within scaffolds. The latter case is due to the fact that the entire scaffold could not be fixed. With histology it was possible to observe that, among scaffolds, SHE (HA-EAbuK-IKVAV) was the best scaffold not only for the highest viability measured (viability assay results), but also the one that allowed faster growth of the adherent cells. On the contrary, SHR1, SHR2, SHR3 (HA-RGD-EAbuK) and SH (HA) scaffolds appeared to be less effective in promoting cell adhesion processes. We attribute the capacity of efficiently interacting with the cell adhesion mechanism to the peptide’s arrangement.

TEM analysis of adherent cells from 4/5 scaffolds confirmed the presence of both intracellular and extracellular collagen microfibrils. For what concerns collagen, procollagens are secreted out of fibroblasts, and after propeptides removal, tropocollagens are formed [62, 63]. Collagen microfibrils are then formed when regular cross-linking of tropocollagens occur in the extracellular space [64]. It is known that a regular cross-striated structure characterizes collagen microfibrils at TEM [65], as we could observe in our findings. Collagen microfibrils were not present in Matrigel, 2D culture and in SH. In regard to the fact that no collagen microfibrils were visible in SH, which is made only by HA, it has been shown that the amount of ECM produced (i.e., the amount of GAG secretion and the expression of collagen gene markers) is affected by the pore size of scaffolds [23]. Moreover, this finding demonstrates the importance of the presence of SAPs to induce the production of ECM components.

By TEM analysis, dilated RER and damaged mitochondria with broken or disrupted cristae were detected both in scaffolds and in Matrigel. In both scaffolds and Matrigel the severity of these signs of cellular suffering increased from 24h to 7 days, but, unlike in scaffolds, in Matrigel at 7 days only cellular remnants were visible indicating a complete cell lysis. The RER is an organelle involved in protein processing, carbohydrate and calcium metabolism, and lipid biogenesis. When there is protein overproduction or misfolding, proteins accumulate in this organelle, resulting in RER stress and dilation [66]. Mitochondria are intracellular organelles whose function includes energy production, reactive oxygen species (ROS) generation, calcium flux, and apoptosis, to maintain cellular homeostasis [67]. Disturbance of mitochondrial-shaping proteins disrupts their cristae shape. Mitochondrial cristae features have been shown to be correlated with changes in function. In particular, changes in cristae number and shape are correlated with respiratory efficiency and cell viability [68]. To our knowledge, there are no specific data describing similar damaging cell effects at TEM on mammalian fibroblasts growing in Matrigel or in the applied scaffolds.

With regard to the ECM production, it is well-known that *in vivo* fibroblasts produce their own pericellular matrix composed largely of collagens, fibrin, fibronectin, proteoglycans, glycosaminoglycans, and matricellular proteins [69]. By RT-PCR the presence of collagen and laminin transcripts was detected in the adherent cells of all the seeded scaffolds, as previously demonstrated also in scaffolds made of electrospun polycaprolactone fibers seeded with human dermal fibroblasts [70]. The presence of elastin was instead detected only in SH and HA-RGD-EAbuK (SHR1 and SHR3) scaffolds. We didn’t include Matrigel in the analyses since with TEM no collagen microfibrils were found and since there weren’t morphologically intact adherent cells at 7 days. According to the literature, the presence of HA should stimulate the production of extracellular elastin [71]. Indeed, it was demonstrated in human and animal models that HA allows up-regulation of type II transforming growth factor-β receptor and connective tissue growth factor, mediating this stimulation [71–73]. However, it has been reported that elastin biosynthesis is highly dependent upon culture conditions [74]. In bovine fibroblasts cultures, it has been demonstrated that it depends on concentrations of fetal calf serum and on cell density [74]. Related to this, it is already known that within 3D systems, the complicated regulation of the distribution of oxygen, nutrients, and waste influences cellular differentiation and tissue homeostasis. Indeed, it depends on the bulk concentration in the media, its diffusion within the gel and its cellular uptake [29, 74]. Based on these considerations, the detection, in our study, of elastin transcripts only in some scaffolds could be explained by the fact that variable interaction with different types of scaffolds can control mRNA synthesis.

Although this study allowed us to identify HA-EAbuK-IKVAV scaffold as the best 3D model in terms of cell viability, growth and ECM components, there are some limitations. Among the major limitations of the study there is the application of only one cell line and results should be indeed tested on additional lines. However, considering the rarity of cetacean-derived *in vitro* models and availability of stabilized cell lines we still considered this work a pyoneristic approach to test new engineered ECMs and their role as good or bad niches for dolphin fibroblasts. Further the stiffness of the scaffolds and Matrigel did not allow fixation and processing for histology, to better evaluate morphological details of in-suspension cells. Fixation of 3D structures embedded in Matrigel with standard formaldehyde (FA) methods dissolves the ECM. This generates some problems for image-based phenotyping: it changes the relative position of the cells in the well, it can change the original morphology of the cells because of the loss of the supporting matrix. Some groups have devised different strategies to address these challenges in the case of organoids within Matrigel fixation, such as fixing the organoids with formaldehyde and subsequent embedding into paraffin [75]. But this was not possible in our case since bottlenose dolphin fibroblasts created little aggregates compared to organoids that were damaged by the fixation attempts. Additionally, advanced whole genome sequencing approaches would also implement the amount of information of cell genetic modification into the different microenvironments. This broad scale sequencing or quantitative PCR might be applied after the better scaffold has been recognized, as in this study, to test outcomes in—for example—ecotoxicological studies in which skin fibroblasts can have a role in the uptake and stocking of exogenous water-dissolved substances. Therefore, we believe that this preliminary study is still valuable as the base of *in vitro* modelling to approach cetacean pathobiology.

## 5. Conclusions

In this study, we evaluate five different 3D scaffolds and Matrigel for the development of cetaceans’ 3D cell cultures, to obtain a more reliable tool than the 2D approach. In these novel scaffolds we reported deposition of ECM around the cells similarly to the *in vivo* fibroblasts. The Scaffold made exclusively of HA, was found to be the least suitable matrix for fibroblasts growth, emphasizing the importance of the SAPs within the scaffold systems. Matrigel was poorly suited to the growth of fibroblasts as well. On the other hand, the HA-EAbuK-IKVAV scaffold resulted as the best 3D model in terms of cell viability, growth and ECM components rich in collagen and laminin. Besides some above-mentioned limitations, the approach and the main findings reported in this study should facilitate the development of new scaffolds for future pathobiological and ecotoxicological investigations in protected species as cetaceans.

## Supporting information

S1 Fig Confocal microscopy of culture grown on SH, SHR2 and SHR3 scaffolds. Representative confocal microscopy of culture grown SH scaffold (a, d), SHR2 scaffold (b, e), and SHR3 scaffold (c, f) at 24h and 72h after seeding. All the figures show a merge of cellBrite and Hoechst staining, 20xair magnification. Bar represents 50 μm.
(TIF)

S2 Fig He of unconditioned scaffolds. HE images of the unconditioned scaffolds at 24h from the seeding 10x magnification. No extracellular filaments were detected. Bar represents 100 μm.
(TIF)

S3 Fig TEM images of adherent cells within scaffolds. Details of TEM images of adherent cells seeded within SH (a, b, c), SHR1 (d, e, f), SHR2 (g, h, i), SHR3 (j, k, l) SHE (m, n, o) at 24h, 72h and 7 days from the seeding. After 72h from the seeding, apart from SH scaffold in which ECM filaments were not evidenced, TEM analysis showed the presence of collagen microfibrils with different length and shape in all the other HA/peptide-based scaffolds.
(TIF)

S4 Fig RT-PCR for the detection of collagen, laminin and elastin RNA expression. Use of RT-PCR for the detection of collagen, laminin and elastin RNA expression in seeded and unconditioned scaffold (blank) and in cells with their medium (2D) at 72 h and 7 days from seeding. Collagen and laminin transcripts were present in all the scaffolds matrices and in the 2D samples (a, b, c, d, e, f). Elastin transcripts were found in the 2D samples in both time points and in SHR3 at 72h, and in SH and SHR1 at 7 days (g). C: (control) RNA from dolphin skin. B: (blank) RNA from unconditioned scaffolds.
(TIF)

S1 Table Raw data of the CellTiter-Glo^®^ 3D Cell Viability Assay. Luminescence raw data of scaffolds and Matrigel bottom and no bottom conditions in the CellTiter-Glo^®^ 3D Cell Viability Assay.
(TIF)

S2 Table Significant p-values of the CellTiter-Glo^®^ 3D Cell Viability Assay. The significant p-values obtained after inferential analyses are reported. In the bottom condition the difference among means is significant between SH and SHE, SHR1 and SHE, SHR3 and SHE at 24h and it is significant between SH and SHE, SHR1 and SHE, SHR2 and SHE, SHR3 and SHE at 72 h. In the no bottom condition the difference among means is significant between SH and SHR1; SH and SHR2; SH and SHR3; SH and SHE at 24h. The difference among means is also significant between Matrigel in the bottom and Matrigel in the no bottom at 72h.
(TIF)

S1 Raw images The original and uncropped gel results. (PDF)
