# Supplementary figures and images for: Bottlenose dolphin (Tursiops truncatus) immortalized fibroblasts on novel 3D in vitro collagen-free scaffolds
Source: PLoS One. 2024 Jun 11;19(6):e0304992. doi: 10.1371/journal.pone.0304992 (PMC11166351; doi:10.1371/journal.pone.0304992)

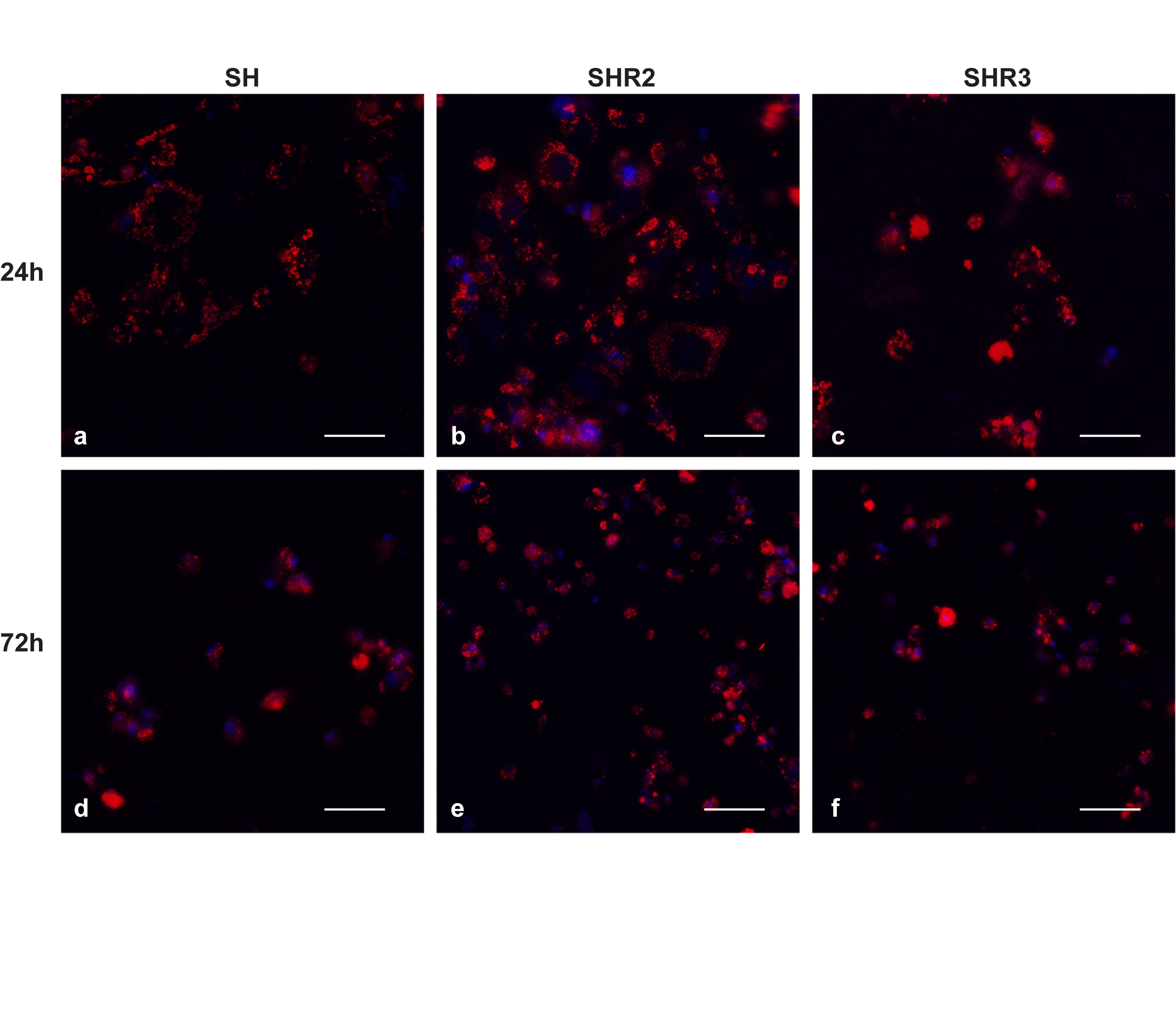

Supplement: S1 Fig — Representative confocal microscopy of culture grown SH scaffold (a, d), SHR2 scaffold (b, e), and SHR3 scaffold (c, f) at 24h and 72h after seeding. All the figures show a merge of cellBrite and Hoechst staining, 20xair magnification. Bar represents 50 μm. (TIF) [file pone.0304992.s001.tif]

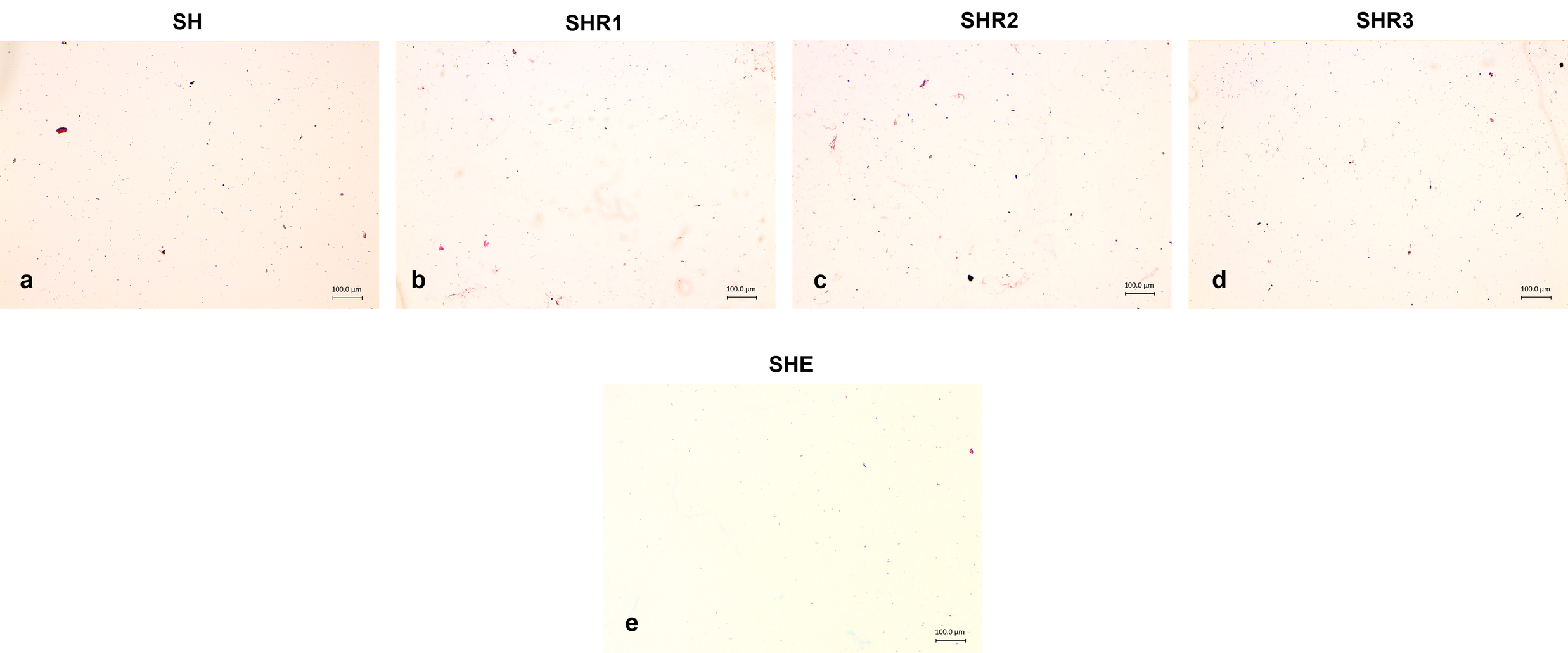

Supplement: S2 Fig — HE images of the unconditioned scaffolds at 24h from the seeding 10x magnification. No extracellular filaments were detected. Bar represents 100 μm. (TIF) [file pone.0304992.s002.tif]

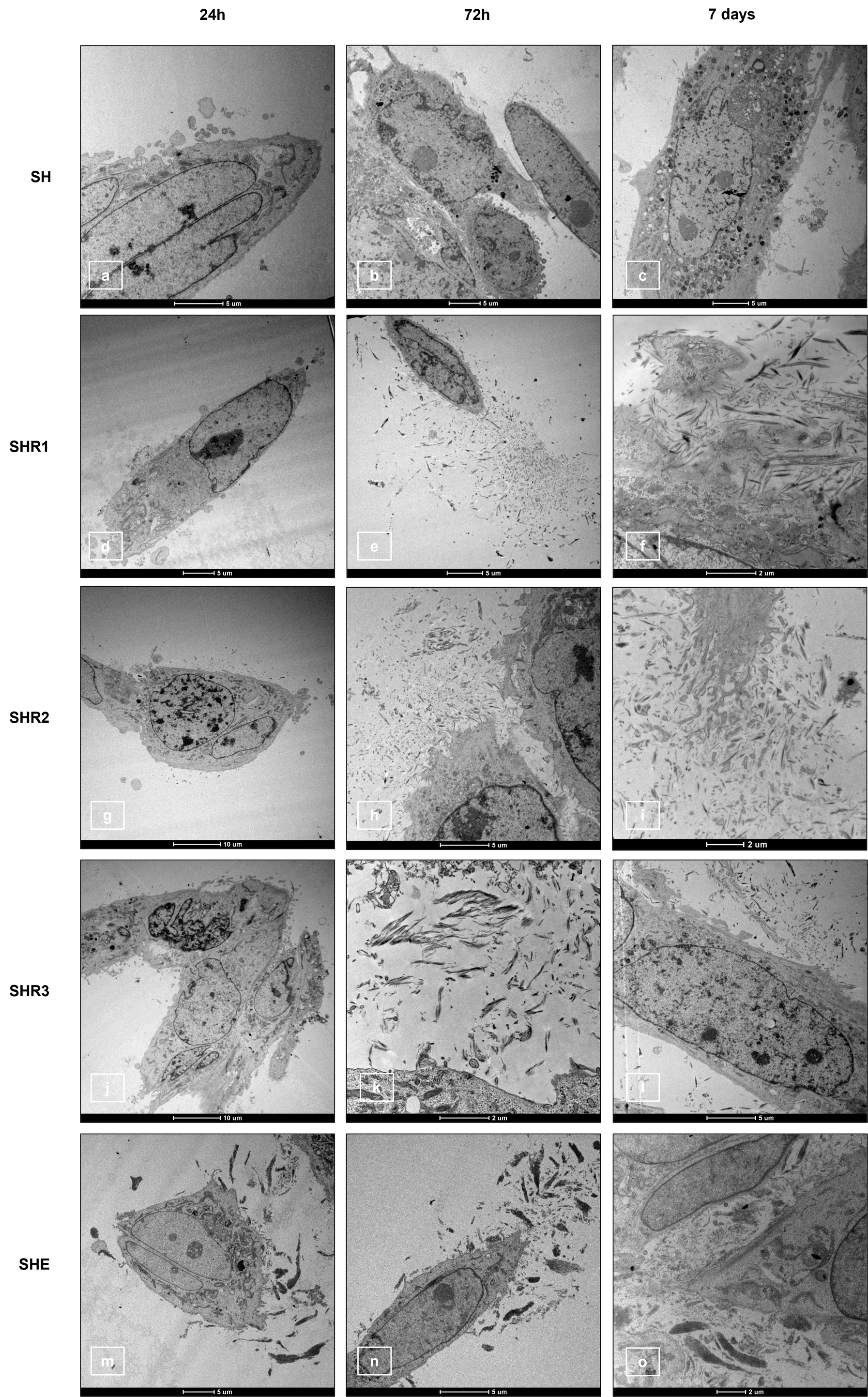

Supplement: S3 Fig — Details of TEM images of adherent cells seeded within SH (a, b, c), SHR1 (d, e, f), SHR2 (g, h, i), SHR3 (j, k, l) SHE (m, n, o) at 24h, 72h and 7 days from the seeding. After 72h from the seeding, apart from SH scaffold in which ECM filaments were not evidenced, TEM analysis showed the presence of collagen microfibrils with different length and shape in all the other HA/peptide-based scaffolds. (TIF) [file pone.0304992.s003.tif]

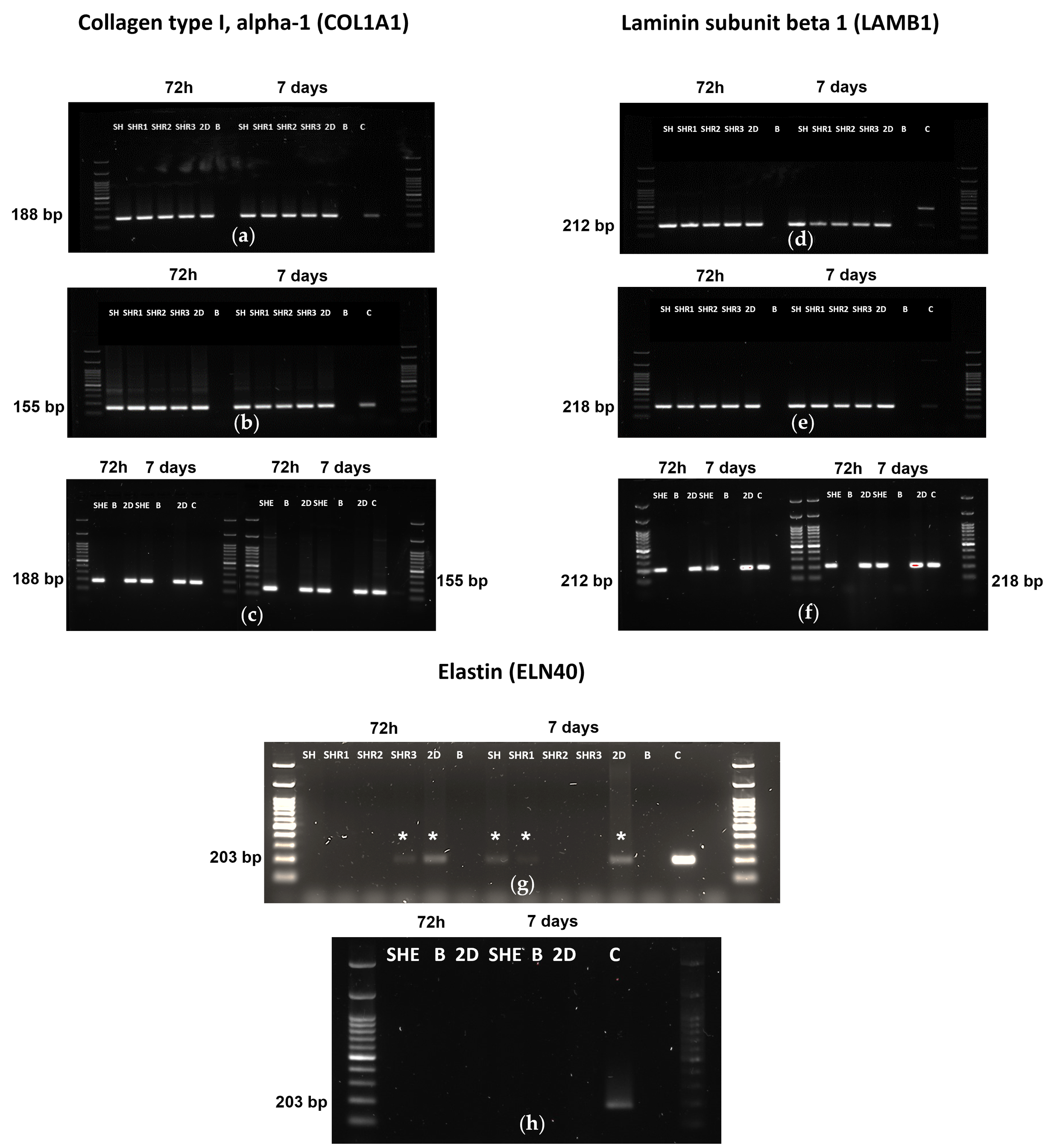

Supplement: S4 Fig — Use of RT-PCR for the detection of collagen, laminin and elastin RNA expression in seeded and unconditioned scaffold (blank) and in cells with their medium (2D) at 72 h and 7 days from seeding. Collagen and laminin transcripts were present in all the scaffolds matrices and in the 2D samples (a, b, c, d, e, f). Elastin transcripts were found in the 2D samples in both time points and in SHR3 at 72h, and in SH and SHR1 at 7 days (g). C: (control) RNA from dolphin skin. B: (blank) RNA from unconditioned scaffolds. (TIF) [file pone.0304992.s004.tif]

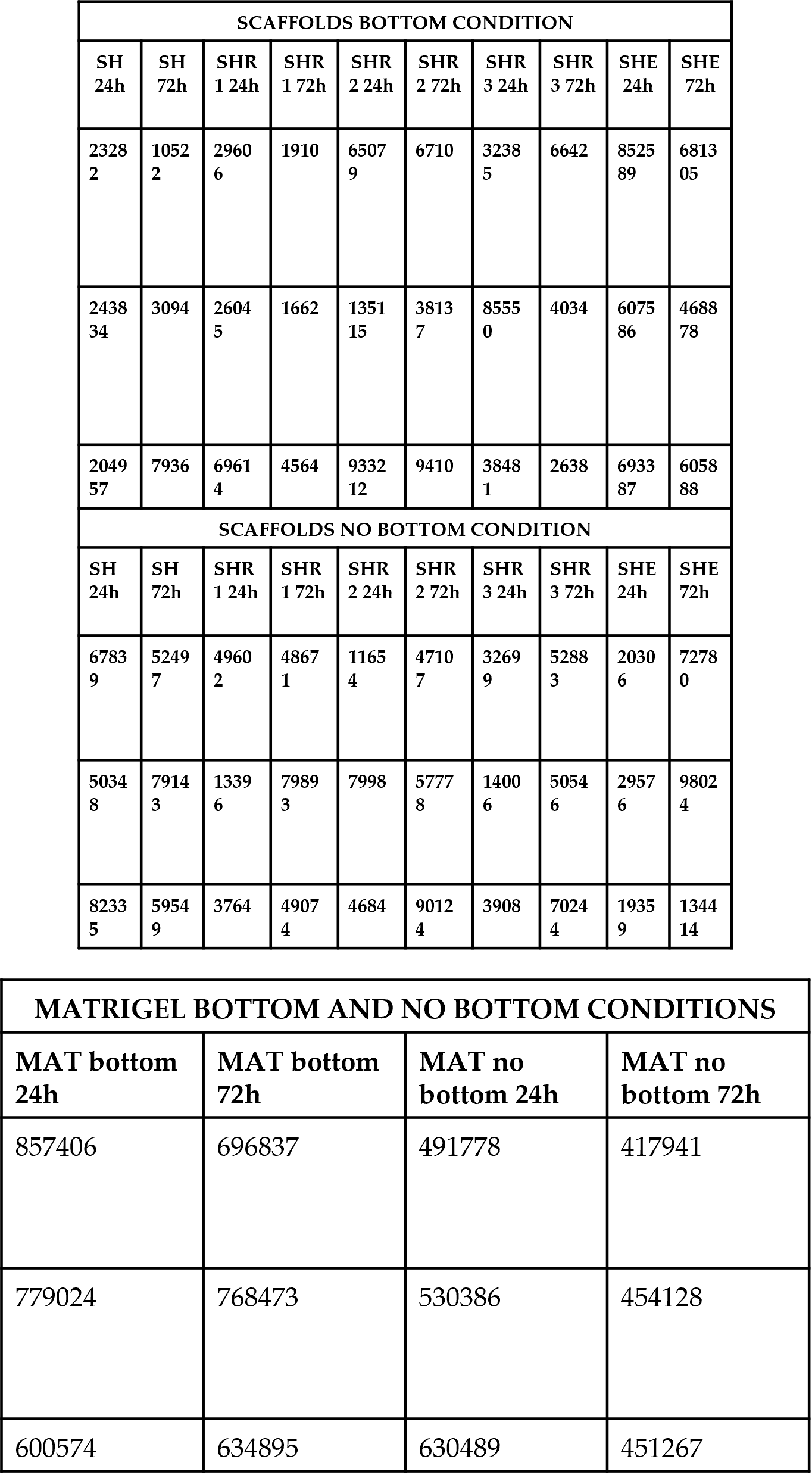

Supplement: S1 Table — Luminescence raw data of scaffolds and Matrigel bottom and no bottom conditions in the CellTiter-Glo® 3D Cell Viability Assay. (TIF) [file pone.0304992.s005.tif]

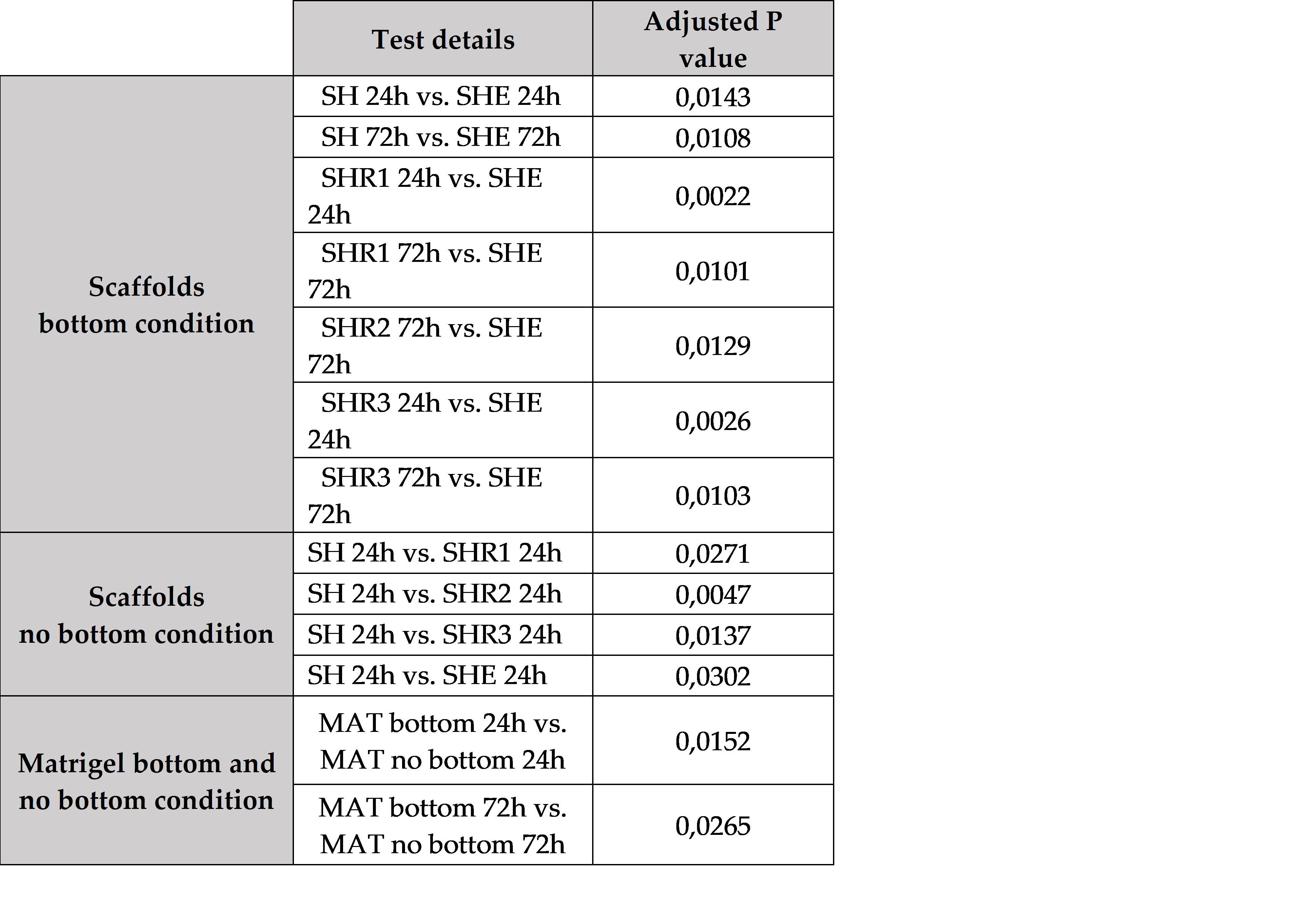

Supplement: S2 Table — The significant p-values obtained after inferential analyses are reported. In the bottom condition the difference among means is significant between SH and SHE, SHR1 and SHE, SHR3 and SHE at 24h and it is significant between SH and SHE, SHR1 and SHE, SHR2 and SHE, SHR3 and SHE at 72 h. In the no bottom condition the difference among means is significant between SH and SHR1; SH and SHR2; SH and SHR3; SH and SHE at 24h. The difference among means is also significant between Matrigel in the bottom and Matrigel in the no bottom at 72h. (TIF) [file pone.0304992.s006.tif]

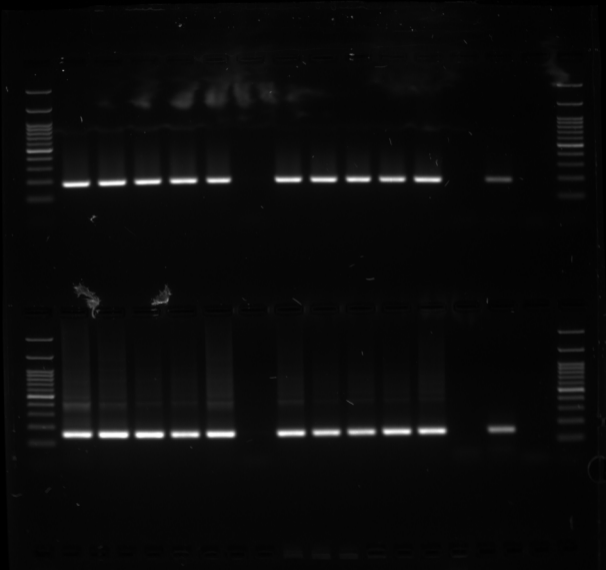

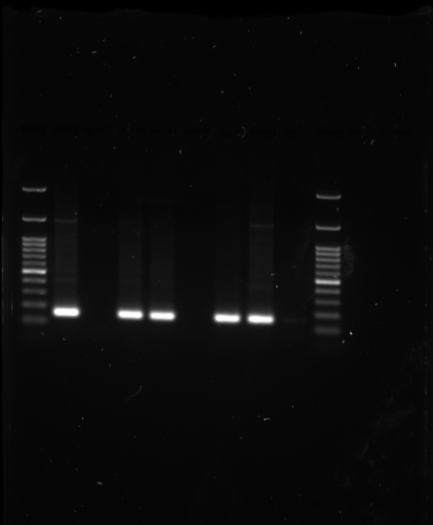

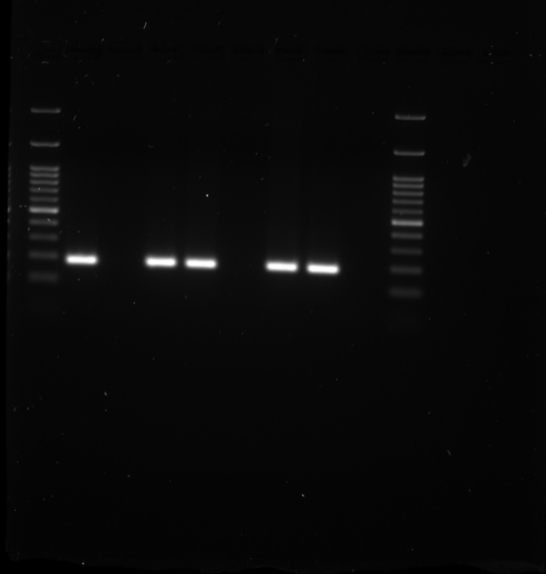

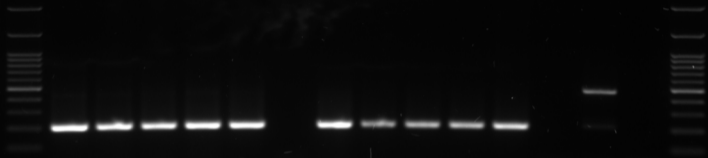

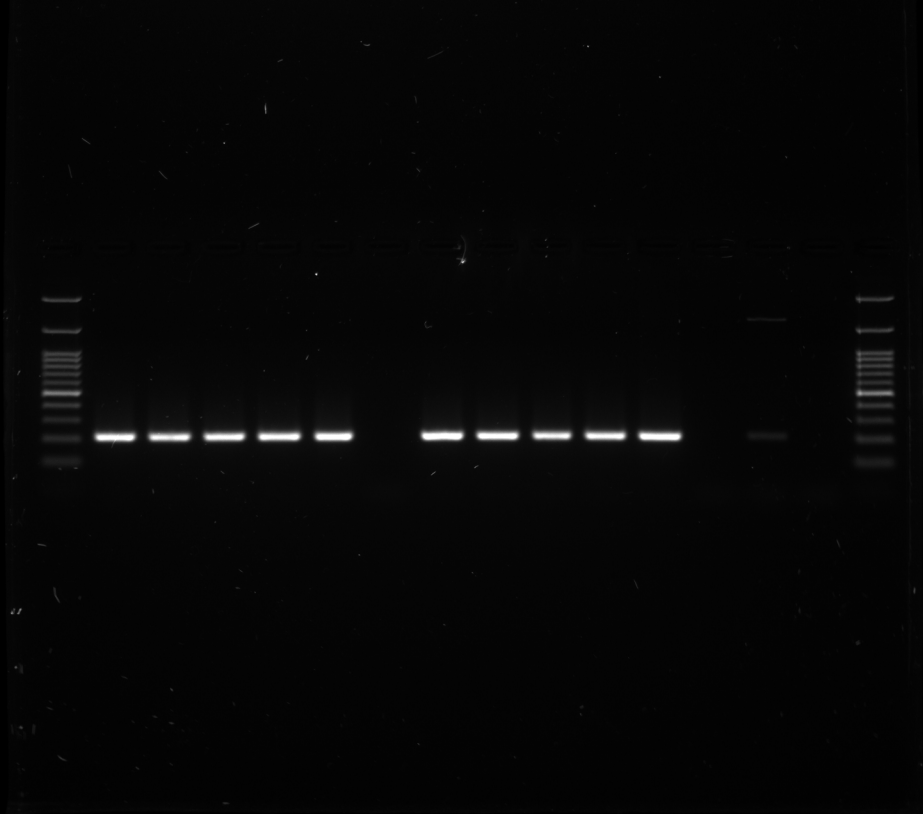

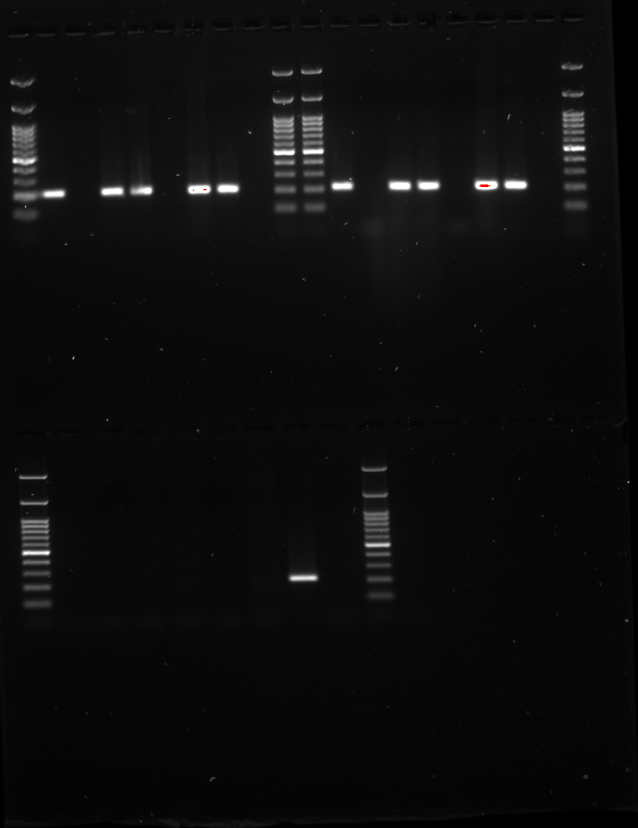

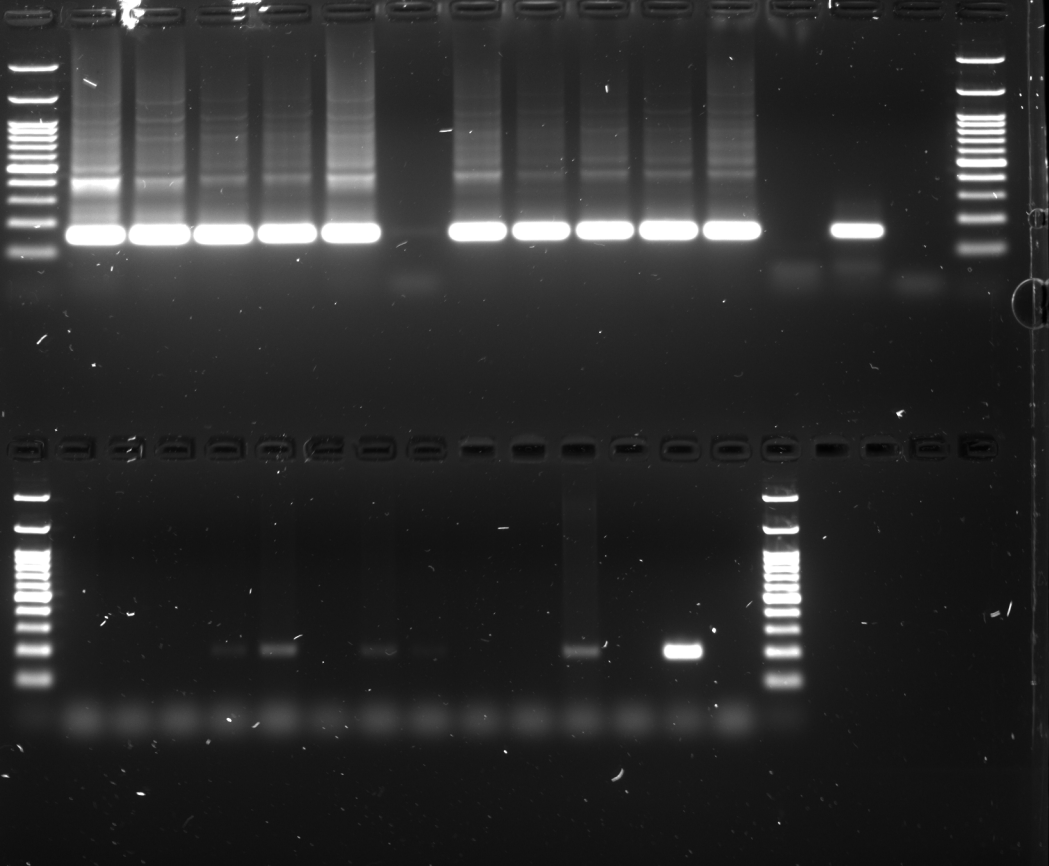

Supplement: S1 Raw images — (PDF) [file pone.0304992.s007.pdf]
